# Impact of Laser Surface Modification on Zirconia Implants: A Systematic Review of In Vitro Evidence

**DOI:** 10.7759/cureus.98697

**Published:** 2025-12-08

**Authors:** Sanjana Bhosale, Arti Gangurde, Manish Chauhan, Niraja Jaiswal, Radha L Harimkar, Sahil Shaikh

**Affiliations:** 1 Prosthodontics and Crown and Bridge, Government Dental College and Hospital, Mumbai, IND

**Keywords:** antimicrobial properties, laser surface modification, osseointegration, wettability, zirconia implants

## Abstract

Zirconia implants have emerged as a viable alternative to titanium due to their superior aesthetics, chemical stability, and biocompatibility. However, their inherently low surface bioactivity has resulted in the development of advanced surface modification techniques to improve osseointegration and biological performance. This systematic review evaluated in vitro studies investigating the effects of laser surface treatment on zirconia implants. A comprehensive electronic and manual search was conducted in PubMed, Cochrane Library, Google Scholar, Scopus, ScienceDirect, and EBSCOhost, including studies published after 2005. Nine in vitro studies met the inclusion criteria. The results demonstrated that laser irradiation significantly enhanced surface roughness, wettability, and protein adsorption while promoting osteoblastic adhesion, proliferation, and differentiation. Femtosecond and Nd:YAG lasers produced well-defined micro- and nano-topographies without inducing deleterious phase transformation or compromising mechanical integrity. Additionally, laser-treated zirconia exhibited reduced bacterial adhesion, suggesting improved antimicrobial potential. Despite methodological variations, all studies indicated positive biological and structural outcomes associated with laser modification. Laser surface modification thus represents a precise, reproducible, and contamination-free approach for enhancing zirconia implant performance.

## Introduction and background

The introduction of zirconia implants has marked a paradigm shift in modern implantology, providing an esthetic and biocompatible alternative to conventional titanium implants. Titanium remains the gold standard owing to its long-term clinical success; however, drawbacks such as corrosion, ion release, and metallic discoloration under thin gingiva have prompted the search for metal-free substitutes [1-3]. Zirconia, a high-strength ceramic, has gained attention due to its great biocompatibility, corrosion resistance, and tooth-like color, making it ideal for patients with metal hypersensitivity or demanding esthetic needs [4]. The earliest in vivo investigation that evaluated zirconia ceramic implants placed in beagle dogs was provided by Akagawa et al. in 1993 [5].

Despite these advantages, zirconia implants exhibit distinct challenges concerning osseointegration, surface bioactivity, and mechanical reliability [6,7]. Unlike titanium, which forms a naturally bioactive oxide layer, zirconia requires surface modification to enhance bone-implant interaction [8,9]. Successful osseointegration depends on surface topography, chemistry, and wettability, which regulate osteoblast adhesion and bone formation [10,11].

To overcome the chemical inertness of zirconia, numerous surface treatments have been explored, including sandblasting, acid etching, plasma spraying, and hydrothermal processing, to increase roughness and bioactivity [12-15]. However, results remain inconsistent because zirconia resists traditional surface alterations [16]. Recently, laser surface treatment has emerged as an auspicious and minimally invasive alternative capable of producing controlled micro- and nanoscale modifications while preserving structural integrity [17,18].

Lasers are widely used in dentistry for soft-tissue surgery, endodontics, and implantology [19,20]. Various laser types, including Er:YAG, Nd:YAG, CO₂, diodes, and femtosecond lasers, have been investigated for modifying zirconia surfaces [21-25]. These lasers can customize surface texture and chemistry to enhance osseointegration. Studies report that laser-modified zirconia increases osteoblast proliferation, bone-to-implant contact, and early healing compared with untreated surfaces [26-29]. Laser treatment also improves hydrophilicity and removes organic contaminants, facilitating protein adsorption and cell attachment [30,31].

In addition to biological benefits, laser treatment provides antimicrobial advantages by reducing bacterial adhesion and biofilm formation, particularly of peri-implant pathogens such as Porphyromonas gingivalis and Streptococcus mutans [32-35]. It may also enhance fatigue resistance, crucial for long-term performance under occlusal stress [36-38]. Nevertheless, outcomes depend on laser parameters such as wavelength, energy, and pulse duration, and excessive energy can induce zirconia phase transformation [39].

Given these opportunities and uncertainties, it is essential to systematically evaluate how laser treatment influences the biological, mechanical, and antimicrobial performance of zirconia implants. This systematic review evaluates available evidence to clarify the clinical potential of laser-modified zirconia implants and guide standardized surface optimization for improved long-term success in implant dentistry.

## Review

Methodology

Study Design and Registration

This systematic review was designed and conducted in accordance with the Preferred Reporting Items for Systematic Reviews and Meta-Analyses (PRISMA) guidelines [40]. The protocol was prospectively registered in the International Prospective Register of Systematic Reviews (PROSPERO) prior to the commencement of the review process (Registration Number: [CRD42025641050]). The objective of this review was to systematically evaluate the influence of laser surface treatments on zirconia implant materials, with a particular focus on osseointegration, biological response, antimicrobial effects, and fatigue behavior.

Search Strategy

A comprehensive and systematic literature search was conducted to identify all relevant studies investigating the effects of laser surface modification on zirconia implants. The electronic databases searched included PubMed, Cochrane Library, Google Scholar, Scopus, ScienceDirect, and EBSCOhost. To ensure completeness, manual searches were also performed using hard copies of relevant journals available within institutional libraries. The search strategy incorporated both Medical Subject Headings (MeSH) and free-text terms combined with Boolean operators such as AND and OR to broaden the scope and capture all potentially relevant articles. The primary search terms included “laser treatment,” “zirconia implant,” “osseointegration,” “biological response,” “fatigue behavior,” and “antimicrobial effect.” The search was restricted to studies published in English after January 2005 to ensure inclusion of contemporary research reflecting current laser technologies and zirconia formulations. Duplicate entries were removed using the Elicit systematic review tool prior to screening.

Eligibility Criteria

The inclusion and exclusion criteria for study selection were established according to the PICOS (Population, Intervention, Comparison, Outcomes and Study) framework, which defined the Population, Intervention, Comparator, Outcomes, and Study design parameters. The review included in vitro experimental studies that evaluated the effects of laser surface treatment on zirconia implant materials. Eligible interventions consisted of the use of various laser systems, such as Er:YAG, Nd:YAG, CO₂, diode, and femtosecond lasers, applied to zirconia surfaces. Studies were required to compare laser-treated specimens with non-laser-treated zirconia or conventionally modified surfaces, including sandblasted or acid-etched samples. Only those studies that assessed one or more key outcomes, including osseointegration, biological response, antimicrobial effects, or fatigue behavior, were selected.

Articles were excluded if they were reviews, case reports, case series, or narrative discussions, or if they did not involve laser treatment of zirconia. Studies lacking a comparator group or failing to report at least one of the pre-specified outcomes were also excluded. Only full-text articles published in English after 2005 were considered. The PICOS-based eligibility framework is summarized in Table 1.

**Table 1:** PICOS framework used for article selection in the present study PICOS: Population, Intervention, Comparison, Outcomes and Study

Component	Inclusion Criteria	Exclusion Criteria
Population	Patients with zirconia implants	Studies not involving zirconia implants or non-human studies
Intervention	Laser treatment of zirconia implants using different laser types (Er:YAG, Nd:YAG, COâ‚‚, diode, femtosecond lasers)	Studies that did not include laser treatment as an intervention
Comparator	Non-laser-treated zirconia implants or other surface treatment methods	Studies without a control group or comparator
Outcome	Osseointegration, biological response, antimicrobial effects, fatigue behavior	Studies that did not assess at least one pre-specified outcome
Study Design	In vivo and in vitro studies published after 2005 in English	Review articles, case reports, case series, narrative reviews, studies published before 2005, and non-English articles

Study Selection Process

The study selection was carried out independently by two reviewers using a two-stage screening process. Initially, titles and abstracts of all retrieved studies were examined to identify potentially relevant articles. Full-text versions of studies meeting the preliminary criteria were then evaluated in detail to confirm eligibility. Disagreements between the reviewers were resolved through discussion and consensus, and when necessary, a third reviewer provided arbitration.

Data Extraction

A standardized data extraction sheet was developed and pre-tested to ensure consistent data collection across all included studies. Two reviewers independently extracted information regarding the author and year of publication, country, study design, sample size, type and composition of zirconia, laser type, irradiation parameters, comparator groups, and primary outcomes. Data pertaining to osseointegration were derived from measures such as bone-to-implant contact or cell adhesion; biological response data included parameters such as cell morphology, viability, and proliferation; antimicrobial effects were assessed through bacterial adhesion or colony-forming unit counts; and mechanical behavior was evaluated using metrics like surface roughness, flexural strength, and fatigue resistance. In cases of discrepancy, data were re-evaluated and verified by consensus, ensuring the reliability and accuracy of extracted information. The final extracted data were tabulated for narrative synthesis and interpretation.

Risk of Bias Assessment

The methodological quality and risk of bias of the included in vitro studies were assessed using the Quality Index for In Vitro Studies (QUIN) tool [41]. This validated instrument evaluates 12 methodological domains, including clarity of research objectives, justification of sample size, presence of appropriate control groups, standardization of experimental protocols, adequacy of surface characterization, assessment of biological parameters, blinding of outcome evaluation, appropriateness of statistical analysis, and transparency in reporting material and laser parameters. Additional domains assess the replicability of methods and whether conclusions were supported by the results obtained. Each domain was scored on a scale of 0 to 2, and cumulative scores determined the level of bias. Studies with total scores of 20 or above were considered to exhibit low risk of bias, those scoring between 14 and 19 were categorized as moderate risk, and studies with scores below 14 were deemed high risk.

Data Synthesis and Analysis

Due to the considerable heterogeneity among the included studies in terms of study design, laser type, irradiation parameters, outcome measures, and analytical methods, a meta-analysis could not be performed. Instead, a qualitative narrative synthesis was undertaken. Extracted data were systematically categorized and analyzed to identify recurring patterns and trends. Comparisons were made based on the type of laser used, specific treatment parameters, and the range of evaluated outcomes, including changes in surface morphology, wettability, osseointegration potential, antimicrobial behavior, and mechanical integrity. This approach facilitated a comprehensive understanding of how various laser systems influence the surface characteristics and biological performance of zirconia implants.

Results

**Figure 1:**
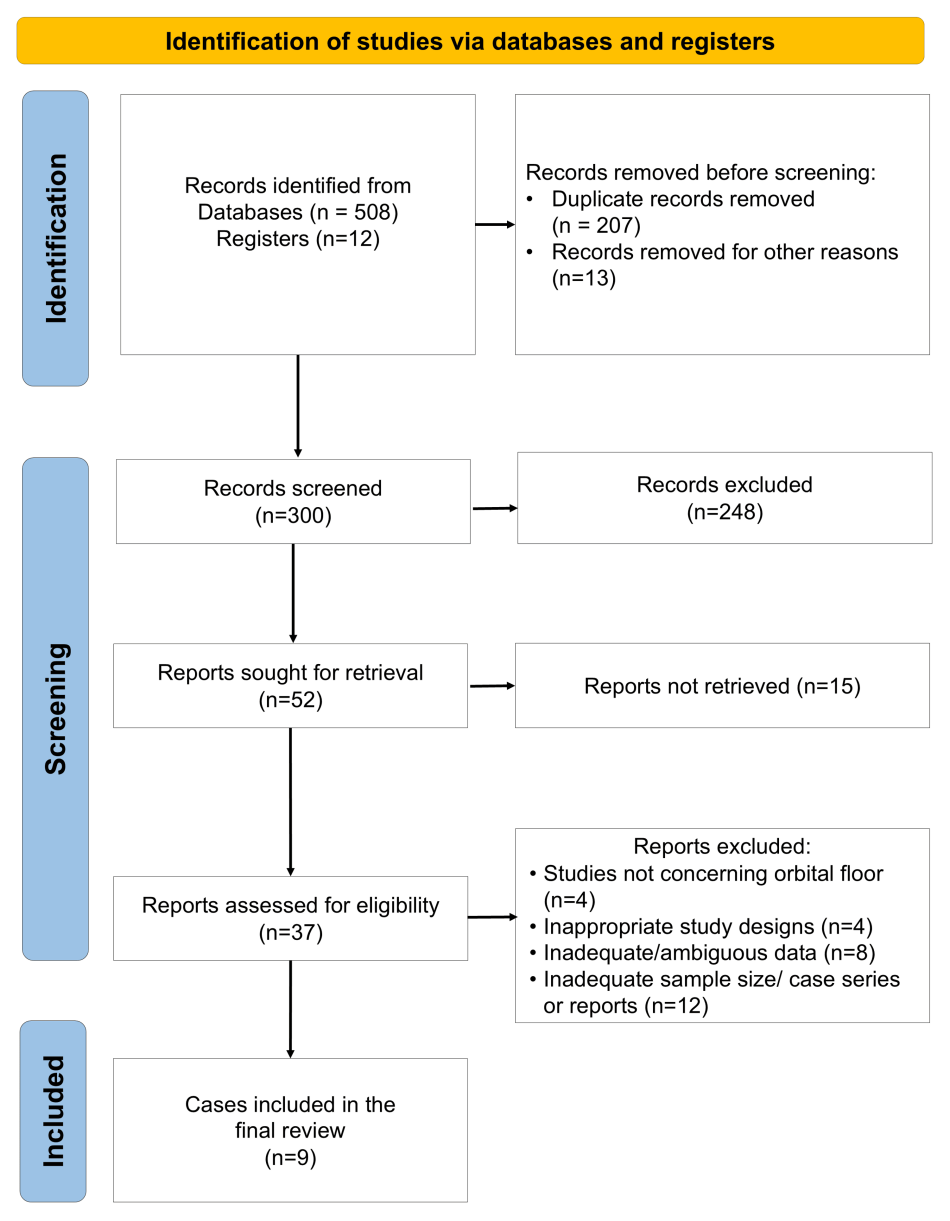
PRISMA flow diagram indicating the article selection process in the present systematic review PRISMA: Preferred Reporting Items for Systematic Reviews and Meta-Analyses

A total of nine in vitro studies met the inclusion criteria and were analyzed in this systematic review (Figure 1) [42-50]. Each study investigated the influence of laser surface treatments on zirconia implant materials, focusing on morphological, mechanical, biological, and antimicrobial outcomes. The extracted data on study characteristics are detailed in Table 2, and those on outcomes are summarized in Table 3 and Table 4.

**Table 2:** Characteristics of the included studies SEM: Scanning electron microscopy; XRD: X-ray diffraction; COF: coefficient of friction

Author(s), Year	Country	Study Design	Sample Size	Type of Zirconia Implant Used	Laser Type	Laser Parameters	Comparator	Outcome Measures
Hauser-Gerspach et al. 2010 [47]	Switzerland	In vitro	≥5 independent experiments per group; 8 disks per bacterial suspension (1 control, 1 CHX, 6 laser)	Y-TZP-MS (microstructured), Y-TZP-POL (polished)	CO₂ (10,600 nm), Diode (810 nm)	CO₂: 2W 10s (100 J/cm²), 4W 60s (1200 J/cm²); Diode: 1W 10s (50 J/cm²), 3W 10s (150 J/cm²); all continuous wave	Untreated zirconia, titanium (T-SLA, T-POL), CHX 2% (30s)	CFU counts of S. sanguinis and P. gingivalis (planktonic and adhered)
Delgado-Ruiz et al. 2010 [48]	Spain	In vitro	66 implants; 22 per group (Control, Pored, Grooved); subsets used for SEM, profilometry, XRD, Raman	White SKY zirconia implants (3Y-TZP)	Femtosecond (Ti:Sapphire, 795 nm, 120 fs)	1 kHz, up to 1.1 mJ; Pores (30 µm dia, 70 µm pitch); Grooves (30 µm wide, 70 µm pitch); 3D rotary laser setup	Sandblasted zirconia implants	SEM, interferometric profilometry, XRD, Raman spectroscopy (surface morphology, roughness, crystallinity)
Moura et al., 2017 [42]	Brazil	In vitro	12 discs (3 per group)	3Y-TZP zirconia discs	Nd:YAG	1064 nm; 0.9 W and 1.8 W; 20 kHz; scan speed 15 mm/s	As-sintered; sandblasted and acid-etched	Friction coefficient; surface roughness; contact angle; phase transformation
Faria et al., 2019 [43]	Portugal	In vitro	Multiple samples per group, min 3 per laser setting	3Y-TZP (green-state compacted)	Nd:YAG (1064 nm)	6W, 35 ns, 0.3–1.5 W, 1–8 passages; spot size 3 μm; scan speed 200 mm/s; 2 strategies (Z8, Z16)	As-sintered (AS) and sandblasted-acid etched (SB-AE)	Surface roughness, contact angle, COF (static/dynamic), monoclinic content (XRD), flexural strength
Carvalho et al. 2019 [45]	Portugal	In vitro	Triplicate experiments per group	Alumina toughened zirconia (80% 3Y-TZP + 20% Al₂O₃)	Femtosecond laser (800 nm, 150 fs)	Scan speed 2 mm/s; grooves (~40 µm spacing); grids formed by cross-scanning	Untreated ATZ ceramic	SEM, profilometry (Ra, Rq, Rp), XRD, wettability, metabolic activity, confocal microscopy
Fernandes et al., 2020 [44]	Portugal	In vitro	65 discs of 3Y-TZP	3Y-TZP (Tosoh Corp., 3 mol% Y2O3)	Nd:YAG laser (1064 nm)	6 W, 30 µm spot, 35 ns, 20 kHz; fluence: 4.95×10⁵ J/cm²; scan speed: 128 mm/s; 1, 2, 4, or 8 passes	SBAE (sandblasted and acid-etched control)	Cell viability (resazurin assay), ALP activity, collagen I (ELISA), SEM morphology
Wawrzyk et al., 2021 [49]	Poland	In vitro	10 zirconia implant	10 zirconia implants 3Y-TZP	Diode laser (Elexxion claros, 810 ±10 nm)	25 W, PM: 15000 Hz, 10 µs pulses, L1: 1×15 s, L2: 2×15 s, L3: 3×15 s with 1 min intervals	Zirconia (untreated vs L1, L2, L3 laser-treated)	Microbial load (CFU), MALDI-TOF, NGS, SEM, profilometry
da Cruz et al., (2022) [46]	Portugal	In vitro	15 discs per group (3 groups: laser, milling, SBAE)	3Y-TZP (Tosoh Corp., 3 mol% Y2O3)	Nd:YAG laser (1064 nm)	6 W, 20 kHz, 2000 mm/s, 30 μm spot, 0.3 mJ/pulse	Laser vs. Milling vs. SBAE (untextured control)	Cell viability, morphology, IL-1β, IL-8, osteopontin, collagen I, ALP
Majidov et al., 2024 [50]	USA	In vitro	3 samples (27% patterned, 36% patterned, control)	Monoclinic ZrO_2_ nanopowder sintered to tetragonal	Nd:YAG (1064 nm)	10 Hz, 5 ns pulse, 3 mm beam, 2 J/cm² fluence	two laser patterned groups compared with untreated controls	Protein adsorption, surface characterization

**Table 3:** Mechanical and biological outcomes across the included studies

Author(s), Year	Surface Roughness	Wettability	Contact Angle	Protein Adsorption	Primary Stability	Biological Response	Cell Adhesion/Spreading	Osteoblast Viability	Other Biological Markers
Hauser-Gerspach et al. 2010 [47]	minimal surface disruption	Not assessed	Not assessed	Not assessed	Not assessed	Not applicable (focus on microbial reduction)	Not assessed	Not assessed	Not assessed
Delgado-Ruiz et al. 2010 [48]	Grooved: ~6× roughness increase; Pored: ~1.2× increase compared to control	Not assessed	Not assessed	Not assessed	Not assessed	Not assessed	Not assessed	Not assessed	Elemental purity: ↑Zr, ↓C/Al in treated surfaces
Moura et al., 2017 [42]	Ra: AS (0.39 µm), LI (1.04 µm), LII (2.82 µm); increased surface irregularity	Contact angle measured; improved surface wettability	AS: 46.9°, SE: 44.6°, LI: 38.2°, LII: 36.4°	Not assessed	COF measured as surrogate; better primary stability	Not assessed	Observed qualitatively by SEM/EDS	Not assessed	Bone adhesion evaluated via SEM/EDS
Faria et al., 2019 [43]	Ra: AS (0.14 μm), SB-AE (2.01 μm), Laser: up to 39.8 μm texture depth ; average roughness features	WCA: 0°–13.2° (laser), 8° (SB-AE), 21.3° (AS) improved hydrophilicity	0°–13.2°	Not assessed	COF ↑ with laser power/passages, max initial COF: 1.09 showing enhanced primary stability	laser structured zirconia supported better cell distibution post sintering	Observed via SEM/EDS	Not assessed	Not assessed
Carvalho et al. 2019 [45]	Ra: Control 0.71 µm; Grooves 2.00 µm; Grids 3.21 µm; surface roughness increased	Increased in laser-treated groups	not assessed	Not assessed	Not assessed	Increased proliferation; enhanced metabolic activity; cytoskeletal alignment and spreading on microstructured surfaces	Spread morphology and alignment along grooves and grids observed in SEM and confocal microscopy	Increased via resazurin assay	not assessed
Fernandes et al., 2020 [44]	Ra: between 1,5,2 µm for all groups (profilometry) found direct correlation between no. of laser passes and surface complexity	Not assessed	Not assessed	Not assessed	Not assessed	Increased cell viability and collagen I vs control; no difference in ALP	Groove textures: elongated veil-like cells; pillar textures: prismatic morphology with apex attachment	Increased viability in all textured groups vs SBAE	Not assessed
Wawrzyk et al., 2021 [49]	Porcelain: Ra decreased (from 2.08 to 0.93 µm); Zirconia: mixed Ra change (0.79 to 1.10 µm depending on scan area); no significant differences were observed in roughness	Not directly measured; but improved	not measured by qualitatively reported improved wettability	Not assessed	Not assessed	Indirect via microbiota and inflammatory marker data	Not assessed	Not applicable	Interleukin/fungal pathogen data via NGS (e.g. M. restricta; Rothia spp.)
da Cruz et al., 2022 [46]	Ra: SBAE 2.25 ± 0.42 μm; Laser 2.23 ± 0.14 μm; Milling 1.13 ± 0.36 μm increased surface area for increased biological interaction	improved	lowest contact angle	Not assessed	Not assessed	Osteoblast: ↑ viability and differentiation in milling compared to laser texturing	Osteoblasts adhered in all groups; Milling showed better orientation; Fibroblasts: flatter and more spread on milling	Osteoblast: Milling > Laser; Fibroblast: Laser > Milling (day 3 only)	IL-1β and IL-8 levels: no significant differences; osteopontin: Milling > Laser
Majidov et al., 2024 [50]	Not numerically reported; micropatterned regions with periodic grooves under SEM	Not directly measured; but improved	green 0, presintered 60, sintered 82; improved wettability	BSA adsorption 92% higher for 27% coverage, 169% for 36%	Not assessed	Increased BSA adsorption indicates improved osseointegration	Supported by SEM and AFM	Not assessed	Not assessed

**Table 4:** Osseointegration-related, microbial, and metallurgical outcomes along with conclusive findings reported by authors of the included studies

Author(s), Year	Gene Expression (e.g. Runx2, ALP, OCN)	BIC or Simulated Osseointegration	Collagen Formation	Osseointegration Outcome	Antimicrobial Effects	Antibacterial Log Reduction	CFU (Colony Forming Units)	Flexural Strength	Mechanical Performance	Phase Transformation	Statistical Significance	Main Findings	Conclusion
Hauser-Gerspach et al. (2010) [47]	Not assessed	Not assessed	Not assessed	Not assessed	CO₂ (high): ~4 log reduction CO₂ at lower setting ; Diode (high): 1–2 log at higher setting effective on both polished and microstructured zirconia; similar to CHX	S. sanguinis: 2–4 log; P. gingivalis: up to 6 log reduction depending on laser	S. sanguinis: 2–4 log reduction; P. gingivalis: up to complete inactivation at high CO₂	Not assessed	Not assessed	Not assessed	Not clearly stated	CO₂ and diode lasers at high settings significantly reduced viable bacteria; low settings less effective; P. gingivalis more sensitive; results comparable to CHX	High-power laser irradiation effectively reduces oral pathogens on zirconia surfaces; CO₂ laser is particularly promising, suggesting potential for peri-implant biofilm management without altering surface integrity.
Delgado-Ruiz et al. (2010) [48]	Not assessed	Not assessed	Not assessed	Not assessed	Not assessed	Not assessed	Not assessed	Not assessed	Not assessed	Minimal monoclinic transformation; mostly tetragonal zirconia preserved	p < 0.005	Femtosecond laser produced clean microstructures (grooves and pores) with precise geometry and nanoscale texture; increased roughness; reduced monoclinic phase content; no structural damage seen	Femtosecond laser microstructuring improves zirconia surface topography while preserving structural and crystallographic integrity; a promising technique for enhancing implant surface properties
Moura et al., 2017 [42]	Not assessed	Not assessed	Not assessed	Not assessed	Not assessed	Not assessed	Not assessed	Not assessed	Static and dynamic COF; post-friction SEM/EDS	Monoclinic content measured by XRD less as compared to control group	p < 0.05	Higher static and dynamic friction with laser-treated zirconia; better bone adhesion with LII; less monoclinic phase than SE	Laser treatment enhanced wettability, surface roughness, and friction characteristics, making it a promising alternative to conventional treatments
Faria et al., 2019 [43]	Not assessed	Not assessed	Not assessed	Not assessed	Not assessed	Not assessed	Not assessed	521–692 MPa (laser); AS: 795 MPa; SB-AE: 858 Mpa shows flexural strength was reduced after laser treatment	Flexural strength (ball-on-3-ball), fracture zone SEM	Monoclinic: AS (0.92%), SB-AE (7.79%), laser: 1.19–3.03%; <25% after aging	p < 0.05	Higher surface roughness, improved wettability, higher initial static COF with deeper textures; most laser settings maintained flexural strength >500 MPa	Laser texturing in green state enables controlled geometry, superhydrophilic surfaces, and enhanced friction for improved primary stability with acceptable mechanical performance
Carvalho et al. (2019) [45]	Not directly measured, osteogenic trends indicated	Not assessed	Not assessed	Not assessed	Not assessed	Not assessed	Not assessed	Not assessed	Not assessed	Minimal monoclinic phase observed in all groups	p < 0.05	Laser-textured surfaces improved roughness, wettability, and cell behavior without structural damage	Femtosecond laser microtexturing is a promising approach for modifying zirconia surfaces to enhance cellular response and surface functionality
Fernandes et al., 2020 [44]	ALP showed no significant difference	Not assessed	not directly assessed ;Collagen I ↑ in all laser groups vs SBAE	Not assessed	Not assessed	Not assessed	Not assessed	Not assessed	Not assessed	Not assessed	p < 0.05 for viability and collagen I vs control	Laser-textured surfaces improved osteoblast viability and collagen production; texture type and spacing influenced morphology	Surface topography influenced cell response; laser-textured 3Y-TZP enhanced biological performance over SBAE
Wawrzyk et al., 2021 [49]	Not assessed	Not assessed	Not assessed	Not assessed	Yes, reduction: up to 99.91%; best at L3 on all surfaces	S. aureus ATCC: 99.94% (tooth); up to 99.87% (zirconia)	Yes, quantified per microorganism and material	Not assessed	No mechanical changes observed	Not assessed	p < 0.05 for most microbial reductions, esp. L3 exposure	Laser irradiation significantly reduced CFU counts (especially L3); no destructive effects on the material surface	Laser irradiation (triplicate 15 s) shows high antimicrobial efficacy on zirconia, porcelain, and tooth surfaces without altering surface integrity
da Cruz et al., (2022) [46]	ALP ↑ over time, no significant group differences	Not assessed	not directly assessed;Osteoblast collagen I: Milling > Laser; Fibroblast collagen I: Laser > Milling (day 3 only)	Not assessed	Not assessed	Not assessed	Not assessed	Not assessed	Not assessed	Not assessed	Osteoblast viability ↑ over time in all groups; Milling > Laser at day 7 and 14; p < 0.05	Surface texturing influences osteoblast and fibroblast responses; milling showed more favorable biological performance overall	Surface topography and method of texturing influence cell behavior; milling supports better osteogenic markers than laser
Majidov et al., 2024 [50]	Not assessed	Not assessed	Not assessed	Indirectly assessed via protein adsorption	Not assessed	Not assessed	Not assessed	Not assessed	Improved mechanical interlocking via deep patterns	Complete monoclinic to tetragonal transition post-sintering	Yes (p < 0.05 for protein adsorption)	Higher pattern density led to increased protein adsorption	Surface patterning improves osseointegration potential

Study Characteristics

The included studies were published between 2010 and 2024, reflecting growing global interest in laser-modified zirconia implants. Portugal contributed to the largest number of studies (n=4) [43-46]. Other investigations originated from Brazil (Moura et al. 2017), Switzerland (Hauser-Gerspach et al. 2010), Spain (Delgado-Ruiz et al. 2011), Poland (Wawrzyk et al. 2021), and the United States (Majidov et al. 2024) [42,47-50]. This wide distribution underscores the multidisciplinary, international pursuit of optimizing zirconia implant surfaces using laser technologies.

All nine investigations adopted in vitro experimental designs involving standardized zirconia discs or implants subjected to controlled laser irradiation [42-50]. Sample sizes varied considerably across studies, depending on their objectives. Hauser-Gerspach et al. used ≥5 replications per group with eight discs per bacterial suspension [47], while Delgado-Ruiz et al. (2010) divided 66 implants into three groups of 22 specimens each [48]. Moura et al. used 12 discs across different energy settings [42], and Fernandes et al. performed laser treatment on 65 3Y-TZP discs [44]. Carvalho et al. and Faria et al. applied triplicate replications per condition [43,45], whereas da Cruz et al. used 15 discs per group across laser-treated, milled, and untreated samples [46]. Wawrzyk et al. incorporated 10 zirconia crowns [49], and Majidov et al. (2024) tested three zirconia samples with varying patterned areas [50]. Each study maintained internal replication to ensure the reliability of the comparative evaluation between laser-treated and control surfaces.

Types of Zirconia Used

Most studies used 3 mol% yttria-stabilized tetragonal zirconia polycrystal (3Y-TZP), the most widely adopted ceramic in implant dentistry due to its balance of strength, toughness, and biocompatibility [42,44,50]. Hauser-Gerspach et al. (2010) compared microstructured and polished 3Y-TZP [47], while Delgado-Ruiz et al. examined standardized “White SKY” 3Y-TZP implants [48]. Moura et al. and Fernandes et al. employed similar compositions to analyze power-dependent alterations [42,44]. Carvalho et al. (2019) investigated alumina-toughened zirconia (ATZ) containing 80 % 3Y-TZP and 20 % alumina, [45] whereas Faria et al. (2019) experimented on green-state compacted zirconia subjected to laser structuring before sintering [43]. Da Cruz et al. (2022) compared laser-treated, milled, and untreated 3Y-TZP discs [46], and Wawrzyk et al. (2021) confirmed the presence of the tetragonal phase typical of 3Y-TZP despite unspecified formulation [49]. Majidov et al. (2024) used partially stabilized zirconia showing a predominant tetragonal phase [50]. Overall, the included studies maintained consistency in material type, enabling valid inter-study comparison.

Laser Systems and Parameters

A wide variety of laser systems were utilized. Three studies (Moura et al. 2017; Faria et al. 2019; Fernandes et al. 2020) [42-44] employed Nd:YAG lasers operating at 1064 nm with power outputs between 0.3 and 6 W and nanosecond pulse durations. These created microscale textures via controlled ablation, with Fernandes et al. studying the cumulative effect of 1-8 laser passes [44]. Femtosecond lasers were used by Delgado-Ruiz et al. (2010) and Carvalho et al. (2019) [45,48], operating at 795-800 nm with 120-150 fs pulse durations to produce micro- and nanoscale grooves and pits. Da Cruz et al. (2022) utilized an Nd:YAG system controlled by SISMA software [46], and Hauser-Gerspach et al. (2010) tested both CO₂ (10,600 nm) and diode (810 nm) lasers for antimicrobial analysis [47]. Wawrzyk et al. (2021) applied a femtosecond laser to full-contour crowns [49], while Majidov et al. (2024) used a diode laser to generate micropatterned regions (27% and 36% coverage) [50]. Collectively, studies demonstrated that laser systems could reproducibly alter zirconia surface micro-architecture without inducing phase instability.

Comparator Surfaces

Each study included a control or comparator group to assess the effects of laser modification [42-50].Most used untreated zirconia as baseline. Some, such as Delgado-Ruiz et al. (2010) and Fernandes et al. (2020)[44, 48], employed sandblasted or acid-etched comparators, whereas Faria et al. (2019) and Moura et al. (2017) included both as-sintered and post-sintered controls [42,43]. Hauser-Gerspach et al. (2010) also compared titanium discs [47], and Majidov et al. (2024) evaluated two laser-patterned coverage levels against untreated surfaces [50]. These comparators provided a robust framework for quantifying the relative performance of laser treatments.

Surface Morphology and Roughness

All studies assessed surface morphology, primarily via scanning electron microscopy (SEM) and profilometric parameters such as Ra, Rq, Rp, and Sdr. Laser treatment consistently enhanced surface roughness compared to controls. Hauser-Gerspach et al. (2010) reported visible surface alterations proportional to energy density [47], and Delgado-Ruiz et al. (2010) achieved well-defined grooves and 30 μm pores, confirming topographical enhancement [48]. Moura et al. (2017) and Fernandes et al. (2020) reported energy-dependent increases in Ra values [42,44], whereas Carvalho et al. (2019) and Faria et al. (2019) demonstrated significant roughness improvement without structural compromise [43,45]. Da Cruz et al. (2022) reported higher Sdr values [46], and Majidov et al. (2024) observed periodic micropatterns associated with increased surface complexity [50]. Overall, all studies confirmed that laser texturing effectively enhances surface roughness and morphology conducive to osseointegration.

Wettability and Contact Angle

Six studies assessed wettability through static contact angle measurements. Moura et al. (2017) reported values of 36.4 °- 38.2 ° for laser-treated zirconia, markedly lower than those of controls [42]. Faria et al. (2019) reported even lower angles (0-13.2°)[43], indicating near-complete wetting and high hydrophilicity. Da Cruz et al. (2022) also observed the lowest contact angles in laser-treated samples compared with milled or untreated discs [46]. Although Carvalho et al. (2019), Wawrzyk et al. (2021), and Majidov et al. (2024) did not quantify the angles [45,49,50], all reported qualitative evidence of enhanced wettability. These findings collectively demonstrate that femtosecond and Nd:YAG lasers significantly improve the hydrophilicity of zirconia surfaces, thereby promoting protein adsorption and early cell attachment.

Primary Stability Indicators

None of the included studies directly measured primary implant stability; however, surrogate markers such as friction coefficients and surface area ratios were reported. Moura et al. (2017) found higher static friction coefficients for laser-textured zirconia [42], and Faria et al. (2019) recorded increased Sdr values correlating with improved mechanical interlocking potential [43]. These findings indirectly suggest that laser-induced topographical complexity may enhance initial mechanical stability.

Biological Response and Cell Behavior

Seven studies investigated biological interactions with laser-modified zirconia. Carvalho et al. (2019) observed enhanced cell adhesion, alignment, and proliferation on grooved surfaces of ATZ ceramics [45] while Faria et al. (2019) confirmed improved cell distribution on green-state-textured zirconia after sintering [43]. Fernandes et al. (2020) and da Cruz et al. (2022) reported increased cell viability and spreading on laser-treated surfaces [44,46], and Majidov et al. (2024) found greater BSA adsorption and osteoblast alignment in patterned regions [50]. Although Wawrzyk et al. (2021) focused primarily on adhesive performance [49], their findings support a higher surface energy, which is favorable for cell interactions. Collectively, these studies highlight that laser-induced microtopography promotes cell organization, adhesion, and metabolic activity, all of which are essential for osseointegration.

Osteogenic and Collagen Markers

Only Carvalho et al. (2019) indirectly assessed osteogenic trends, noting cell morphology suggestive of differentiation and early matrix formation [45], whereas Fernandes et al. (2020) and da Cruz et al. (2022) observed variable ALP and collagen I expression without statistical significance [44,46]. Although collagen formation was not directly quantified, SEM observations across studies implied early extracellular matrix deposition on laser-modified surfaces.

Antimicrobial Effects

Two studies examined bacterial adhesion on laser-treated zirconia. Hauser-Gerspach et al. (2010) demonstrated 2-6 log reductions in Streptococcus sanguinis and Porphyromonas gingivalis colonization after CO₂ and diode laser treatment [47], while Wawrzyk et al. (2021) qualitatively observed fewer colonies on laser-patterned crowns [49]. These findings underscore the potential of laser surface modification in minimizing biofilm formation and peri-implant infection risk.

Mechanical Performance and Flexural Strength

Mechanical integrity was evaluated in three studies. Faria et al. (2019) reported flexural strengths of 521-692 MPa for laser-treated zirconia [43], slightly lower than as-sintered (795 MPa) and sandblasted-acid-etched controls (858 MPa) but still within clinical limits. Da Cruz et al. (2022) and Carvalho et al. (2019) found that laser treatment did not compromise frictional resistance or induce microcracks [45,46], and Fernandes et al. (2020) confirmed intact surface integrity even after multiple laser passes [44]. Thus, controlled laser exposure preserves mechanical durability while improving biological properties.

Phase Transformation

Five studies analyzed crystalline phase stability via X-ray diffraction. Delgado-Ruiz et al. (2010) and Carvalho et al. (2019) found negligible monoclinic transformation post-femtosecond laser application [45,48] while Faria et al. (2019) reported only 1.38 % monoclinic content compared to 7.79 % in sandblasted specimens [43]. Moura et al. (2017) similarly observed no significant phase change after Nd:YAG exposure [42]. Overall, laser treatments preserved the dominant tetragonal phase, indicating minimal risk of thermally induced degradation.

Risk of Bias Assessment

Risk of bias was evaluated using the QUIN tool (Table 5), which rates the 12 domains of methodological quality [41]. Six studies, Hauser-Gerspach et al. (2010), Delgado-Ruiz et al. (2010), Carvalho et al. (2019), Faria et al. (2019), Fernandes et al. (2020), and da Cruz et al. (2022), were classified as low risk of bias, while three, Moura et al. (2017), Wawrzyk et al. (2021), and Majidov et al. (2024), were moderate risk [42-50]. No study was categorized as high risk. Common limitations included inadequate blinding and insufficient justification of the sample size, but overall methodological rigor was acceptable, with 2/3 demonstrating low risk.

**Table 5:** Risk of bias assessment using the QUIN tool QUIN: Quality Index for In Vitro Studies

Author(s), Year	Clear Aim	Sample Size Justified	Control Group Used	Standardized Protocol	Surface Characterization	Biological Assessment	Blinding of Outcome	Statistical Analysis	Laser Parameters Reported	Material Properties Reported	Replicability of Methods	Conclusions Supported	Total Score	Risk of Bias
Moura et al., 2017 [42]	2	1	2	1	2	1	0	2	2	2	2	2	19	Moderate
Faria et al., 2019 [43]	2	1	2	2	2	2	0	2	2	2	2	2	21	Low
Fernandes et al., 2020 [44]	2	1	2	2	2	2	0	2	2	2	2	2	21	Low
Carvalho et al. (2019) [45]	2	2	2	2	2	2	1	2	2	2	2	2	23	Low
da Cruz et al., (2022) [46]	2	2	2	2	2	2	1	2	2	2	2	2	23	Low
Hauser-Gerspach et al. (2010) [47]	2	1	2	2	2	2	0	2	2	2	2	2	21	Low
Delgado-Ruiz et al. (2010) [48]	2	2	2	2	2	1	1	2	2	2	2	2	22	Low
Wawrzyk et al., 2021 [49]	2	1	2	1	1	1	0	1	2	2	1	2	16	Moderate
Majidov et al., 2024 [50]	2	1	2	1	2	1	1	1	2	2	1	2	18	Moderate

Summary of Key Findings

Across the nine in vitro studies, laser surface treatment consistently enhanced surface roughness, wettability, and cellular response of zirconia while maintaining mechanical integrity and phase stability. Femtosecond and Nd:YAG lasers proved most effective, producing micro- and nano-structured, hydrophilic surfaces that supported osteoblast adhesion and reduced microbial colonization [42-50]. Although flexural strength marginally decreased in some studies, values remained clinically acceptable. Collectively, the evidence supports laser modification as a reliable and reproducible approach for improving zirconia implant performance while preserving material stability.

Discussion

All included studies employed in vitro experimental designs, allowing precise control over environmental and procedural variables and enabling clear evaluation of how laser parameters influence zirconia surfaces [42-50]. While this enhances internal validity, the lack of in vivo biological complexity limits generalizability, as factors such as immune activity, vascularization, and functional loading cannot be replicated fully in vitro. Nevertheless, such laboratory models remain essential for understanding baseline surface responses and optimizing treatment parameters before advancing to clinical research.

Sample sizes varied widely, from small exploratory groups to larger experiments with multiple discs or implant analogs [42,46-48]. Although smaller studies offered preliminary insights, larger studies provided greater statistical reliability and reduced distortion of effect sizes, supporting stronger conclusions about surface changes and biological behavior [42-50]. Most studies used standardized zirconia discs or implant cylinders, thereby minimizing variability in curvature or porosity and improving comparability of surface roughness and contact angle measurements [42-50]. Some investigations also evaluated green-state zirconia to facilitate easier laser structuring prior to sintering, improving micro-pattern precision [20].

Most experiments used 3Y-TZP, reflecting its dominance in clinical implantology because of its strength, stability, and biocompatibility [42,44]. Additional materials such as ATZ and green-state zirconia were included in some studies, confirming that laser modification is adaptable across multiple zirconia formulations without inducing harmful phase transformations or structural defects [43,45].

Laser type and parameter selection were critical determinants of outcomes. Nd:YAG lasers were frequently used for controlled micro-ablation on dense ceramics [42-44], whereas femtosecond lasers produced highly defined micro- and nano-scale textures through cold ablation with negligible thermal damage [45,46]. CO₂ and diode lasers demonstrated utility for antibacterial effects and surface cleaning [47]. Across all systems, careful calibration of wavelength, pulse duration, and energy prevented overheating or microcracking while enabling desirable surface architecture.

Laser irradiation consistently increased surface roughness and complexity, as confirmed by SEM and profilometric data, which revealed grooves, ridges, and pits that promoted higher surface energy and improved protein adsorption [42-50]. Enhanced wettability was also widely reported, with notable reductions in contact angle, reflecting increased hydrophilicity that supports early cell attachment and accelerated osseointegration [42-44]. Importantly, laser-induced hydrophilicity appears more stable than chemically treated surfaces and may resist degradation with aging or handling [51-55].

Biologically, the reviewed studies demonstrated improved osteoblast adhesion, spreading, viability, and maturation on laser-treated zirconia compared with untreated controls [43-46]. Elevated expression of osteogenic markers and ECM organization consistent with early differentiation were observed, attributable to micro- and nano-features that mimic the native extracellular matrix and activate osteogenic signaling pathways [45,51]. Only two studies assessed antimicrobial behavior, yet both reported reduced bacterial adhesion and biofilm presence on laser-modified zirconia, indicating potential dual benefits for osseointegration and peri-implant disease prevention [47,49]. Mechanical and structural assessments showed that flexural strength, fatigue resistance, and crystalline stability were preserved following laser treatment, with XRD confirming maintenance of the tetragonal phase and minimal monoclinic transformation [43-46]. These findings indicate that properly calibrated laser parameters do not compromise zirconia’s mechanical durability.

Overall, the synthesized evidence demonstrates that laser surface modification is a reliable, reproducible, and contamination-free method for enhancing roughness, hydrophilicity, and biological compatibility of zirconia while maintaining mechanical and structural integrity. Laser-treated zirconia shows clear promise for improved osseointegration and reduced bacterial colonization, although long-term in vivo and clinical studies are necessary to translate these laboratory findings into standardized clinical protocols for implant dentistry.

Limitations and future scope

The primary limitation of this systematic review is that all included evidence was derived exclusively from in vitro studies, which, despite offering high experimental control, cannot fully replicate the dynamic biological environment in vivo where immune activity, vascularization, saliva, microbiota, and functional loading influence implant behavior. Considerable heterogeneity existed across studies regarding laser types, energy settings, number of passes, zirconia formulations, and analytical methods, making direct comparisons challenging and preventing quantitative synthesis. Several studies also lacked standardized reporting of sample size rationale, blinding, or long-term aging procedures, limiting extrapolation to clinical timelines. Furthermore, only a small subset evaluated antimicrobial performance or mechanical fatigue behavior, leaving important clinical parameters underexplored. Future research should therefore prioritize well-designed animal and human studies to validate laboratory findings under physiological loading conditions, establish optimal and safe laser parameter windows, and evaluate long-term effects on osseointegration, peri-implant tissue health, fatigue resistance, and biofilm dynamics. Development of standardized laser protocols and head-to-head comparisons across laser systems, zirconia grades, and adjunctive surface treatments will also be critical for guiding clinical adoption and ensuring reproducible outcomes.

## Conclusions

Within the limitations of this systematic review, laser surface modification was found to significantly enhance the surface characteristics and biological behavior of zirconia implants. The treatment improved surface roughness, wettability, and osteoblastic response without compromising mechanical integrity or inducing unfavorable phase transformations. Femtosecond and Nd:YAG lasers, in particular, demonstrated precise and reproducible alterations that promoted superior cellular and antimicrobial performance. Overall, laser surface treatment represents a safe, controllable, and clinically useful technique for optimizing zirconia implant integration and long-term success.

## References

[1] Lorusso F, Noumbissi S, Francesco I, Rapone B, Khater AG, Scarano A (2020). Scientific trends in clinical research on zirconia dental implants: a bibliometric review. Materials (Basel).

[2] Kim KT, Eo MY, Nguyen TT, Kim SM (2019). General review of titanium toxicity. Int J Implant Dent.

[3] Bashutski JD, Wang HL (2007). Common implant esthetic complications. Implant Dent.

[4] Gautam C, Joyner J, Gautam A, Rao J, Vajtai R (2016). Zirconia based dental ceramics: structure, mechanical properties, biocompatibility and applications. Dalton Trans.

[5] Akagawa Y, Ichikawa Y, Nikai H, Tsuru H (199369). Interface histology of unloaded and early loaded partially stabilized zirconia endosseous implant in initial bone healing. J Prosthet Dent.

[6] Wenz HJ, Bartsch J, Wolfart S, Kern M (2008). Osseointegration and clinical success of zirconia dental implants: a systematic review. Int J Prosthodont.

[7] Dong H, Liu H, Zhou N, Li Q, Yang G, Chen L, Mou Y (2020). Surface modified techniques and emerging functional coating of dental implants. Coatings.

[8] Stich T, Alagboso F, Křenek T, Kovářík T, Alt V, Docheva D (2022). Implant-bone-interface: reviewing the impact of titanium surface modifications on osteogenic processes in vitro and in vivo. Bioeng Transl Med.

[9] Schünemann FH, Galárraga-Vinueza ME, Magini R (2019). Zirconia surface modifications for implant dentistry. Mater Sci Eng C Mater Biol Appl.

[10] Parithimarkalaignan S, Padmanabhan TV (2013). Osseointegration: an update. J Indian Prosthodont Soc.

[11] Kumar PS, KS SK, Grandhi VV, Gupta V (2019). The effects of titanium implant surface topography on osseointegration: literature review. JMIR Biomed Eng.

[12] Zhu G, Wang G, Li JJ (2021). Advances in implant surface modifications to improve osseointegration. Mater Adv.

[13] Rudawska A, Danczak I, Müller M, Valasek P (2016). The effect of sandblasting on surface properties for adhesion. Int J Adhes Adhes.

[14] Casucci A, Osorio E, Osorio R, Monticelli F, Toledano M, Mazzitelli C, Ferrari M (2009). Influence of different surface treatments on surface zirconia frameworks. J Dent.

[15] El-Shrkawy ZR, El-Hosary MM, Saleh O, Mandour MH (2016). Effect of different surface treatments on bond strength, surface and microscopic structure of zirconia ceramic. Future Dent J.

[16] Han MK (2024). Advances and challenges in zirconia-based materials for dental applications. J Korean Ceram Soc.

[17] Saran R, Ginjupalli K, George SD, Chidangil S, V K U (2023). LASER as a tool for surface modification of dental biomaterials: a review. Heliyon.

[18] Marinelli G, Inchingolo AM, Carone C (2024). Laser technology in periodontics. Oral Implantol.

[19] Sachelarie L, Cristea R, Burlui E, Hurjui LL (2024). Laser technology in dentistry: from clinical applications to future innovations. Dent J (Basel).

[20] Romanos GE, Gupta B, Yunker M, Romanos EB, Malmstrom H (2013). Lasers use in dental implantology. Implant Dent.

[21] Matsuyama T, Aoki A, Oda S, Yoneyama T, Ishikawa I (2003). Effects of the Er:YAG laser irradiation on titanium implant materials and contaminated implant abutment surfaces. J Clin Laser Med Surg.

[22] Bussoli M, Desai T, Batani D, Gakovic B, Trtica M (2008). Nd:YAG laser interaction with titanium implant surfaces for medical applications. Radiat Eff Defects Solids.

[23] Cobb C, Vitruk P (2015). Effectiveness of a super-pulsed CO₂ laser for removal of biofilm from three different types of implant surfaces: an in vitro study. Implant Pract.

[24] Stübinger S, Homann F, Etter C, Miskiewicz M, Wieland M, Sader R (2008). Effect of Er:YAG, CO(2) and diode laser irradiation on surface properties of zirconia endosseous dental implants. Lasers Surg Med.

[25] Vorobyev AY, Guo C (2007). Femtosecond laser structuring of titanium implants. Appl Surf Sci.

[26] Cunha W, Carvalho O, Henriques B, Silva FS, Özcan M, Souza JC (2022). Surface modification of zirconia dental implants by laser texturing. Lasers Med Sci.

[27] Berni M, Brancato AM, Torriani C (2023). he role of low-level laser therapy in bone healing: systematic review. Int J Mol Sci.

[28] Gnanamuthu DS (1980). Laser surface treatment. Opt Eng.

[29] Andrukhov O, Huber R, Shi B (2016). Proliferation, behavior, and differentiation of osteoblasts on surfaces of different microroughness. Dent Mater.

[30] Yilbas BS (2015). Laser treatment of zirconia surface for improved surface hydrophobicity. J Alloys Compd.

[31] Peavy GM (2002). Lasers and laser-tissue interaction. Vet Clin Small Anim.

[32] Kreisler M, Kohnen W, Marinello C, Schoof J, Langnau E, Jansen B, d'Hoedt B (2003). Antimicrobial efficacy of semiconductor laser irradiation on implant surfaces. Int J Oral Maxillofac Implants.

[33] Tallarico M, Canullo L, Caneva M, Özcan M (2017). Microbial colonization at the implant-abutment interface and its possible influence on periimplantitis: a systematic review and meta-analysis. J Prosthodont Res.

[34] Jaeggi M, Gyr S, Astasov-Frauenhoffer M, Zitzmann NU, Fischer J, Rohr N (2022). Influence of different zirconia surface treatments on biofilm formation in vitro and in situ. Clin Oral Implants Res.

[35] D'Agostino A, Tana F, Ettorre A (2021). Mesoporous zirconia surfaces with anti-biofilm properties for dental implants. Biomed Mater.

[36] Rosentritt M, Preis V, Behr M, Strasser T (2020). Fatigue and wear behaviour of zirconia materials. J Mech Behav Biomed Mater.

[37] Coray R, Zeltner M, Özcan M (2016). Fracture strength of implant abutments after fatigue testing: a systematic review and a meta-analysis. J Mech Behav Biomed Mater.

[38] Li W, Ding Q, Sun F (2023). Fatigue behavior of zirconia with microgrooved surfaces produced using femtosecond laser. Lasers Med Sci.

[39] Hallmann L, Ulmer P, Wille S (2016). Effect of surface treatments on the properties and morphological change of dental zirconia. J Prosthet Dent.

[40] Tugwell P, Tovey D (2021). PRISMA 2020. J Clin Epidemiol.

[41] Tran L, Tam DN, Elshafay A, Dang T, Hirayama K, Huy NT (2021). Quality assessment tools used in systematic reviews of in vitro studies: a systematic review. BMC Med Res Methodol.

[42] Moura CG, Pereira R, Buciumeanu M, Carvalho O, Bartolomeu F, Nascimento R, Silva FS (2017). Effect of laser surface texturing on primary stability and surface properties of zirconia implants. Ceram Int.

[43] Faria D, Madeira S, Buciumeanu M, Silva FS, Carvalho O (2020). Novel laser textured surface designs for improved zirconia implants performance. Mater Sci Eng C Mater Biol Appl.

[44] Fernandes BF, da Cruz MB, Marques JF (2020). Laser Nd:YAG patterning enhance human osteoblast behavior on zirconia implants. Lasers Med Sci.

[45] Carvalho A, Grenho L, Fernandes MH, Daskalova A, Trifonov A, Buchvarov I, Monteiro FJ (2020). Femtosecond laser microstructuring of alumina toughened zirconia for surface functionalization of dental implants. Ceram Int.

[46] da Cruz MB, Marques JF, Marques AF (2022). Modification of zirconia implant surfaces by Nd:YAG laser grooves: does it change cell behavior?. Biomimetics (Basel).

[47] Hauser-Gerspach I, Vadaszan J, Deronjic I (2012). Influence of gaseous ozone in peri-implantitis: bactericidal efficacy and cellular response. An in vitro study using titanium and zirconia. Clin Oral Investig.

[48] Delgado-Ruíz RA, Calvo-Guirado JL, Moreno P (2011). Femtosecond laser microstructuring of zirconia dental implants. J Biomed Mater Res B Appl Biomater.

[49] Wawrzyk A, Łobacz M, Adamczuk A, Sofińska-Chmiel W, Wilczyński S, Rahnama M (2021). The use of a diode laser for removal of microorganisms from the surfaces of zirconia and porcelain applied to superstructure dental implants. Microorganisms.

[50] Majidov I, Allamyradov Y, Kylychbekov S, Khuzhakulov Z, Er AO (2025). Phase transition and controlled zirconia implant patterning using laser-induced shockwaves. Appl Sci.

[51] Saeidnia S, Manayi A, Abdollahi M (2015). From in vitro experiments to in vivo and clinical studies: pros and cons. Curr Drug Discov Technol.

[52] Chen S, Guo Y, Liu R (2018). Tuning surface properties of bone biomaterials to manipulate osteoblastic cell adhesion and the signaling pathways for the enhancement of early osseointegration. Colloids Surf B Biointerfaces.

[53] Xu LC, Siedlecki CA (2007). Effects of surface wettability and contact time on protein adhesion to biomaterial surfaces. Biomaterials.

[54] Lang NP, Salvi GE, Huynh-Ba G, Ivanovski S, Donos N, Bosshardt DD (2011). Early osseointegration to hydrophilic and hydrophobic implant surfaces in humans. Clin Oral Implants Res.

[55] Wang ZK, Zheng HY, Lim CP, Lam YC (2009). Polymer hydrophilicity and hydrophobicity induced by femtosecond laser irradiation. Appl Phys Lett.

